# Application value of halo‑pelvic traction in the treatment of severe rigid spinal deformity

**DOI:** 10.1007/s43390-025-01184-y

**Published:** 2025-09-12

**Authors:** Changlin Lv, Ziang Zhang, Xuanyu Dong, Jianyi Li, Jianwei Guo, Tianyu Bai, Xiaofan Du, Guodong Zhang, Jiale Shao, Jiayan Li, Yukun Du, Jun Dong, Guodong Wang, Yongming Xi

**Affiliations:** 1 The Affiliated Hospital of Qingdao University, No. 59 Haier Road, Laoshan District, Qingdao, 266100 Shandong Province China; 2 Department of Spinal Surgery, Tengzhou Central People’s Hospital, Tengzhou, Shandong Province China; 3 Department of Spinal Surgery, Shandong Provincial Hospital Affiliated to Shandong First Medical University, Jinan, China

**Keywords:** Severe rigid spinal deformity, Halo‐pelvic traction, Halo‐gravity traction, Pulmonary function, Osteotomy grade

## Abstract

**Purpose:**

Comparison of the clinical outcomes of halo-gravity traction (HGT) and halo-pelvic traction (HPT) was performed in the treatment of patients with severe rigid spinal deformity, with the aim of elucidating the clinical value of HGT and HPT in managing such deformities and providing evidence-based recommendations for surgical treatment planning.

**Methods:**

A retrospective study was conducted of 20 patients treated at two large tertiary hospitals (2019–2022). All underwent posterior osteotomy correction and were categorized into HGT (*n = *14) and HPT (*n = *6) groups. Key parameters analyzed included radiographic measures (Cobb angles), pulmonary function tests (before and after traction/surgery), and intraoperative metrics such as blood loss, surgery duration, and osteotomy grade. Health-related quality of life was evaluated using the SRS-22 questionnaire.

**Results:**

Baseline characteristics were comparable between groups. Compared to the HPT group, the HGT group showed significantly lower correction rates in both coronal and sagittal Cobb angles (*P < *0.01), longer surgical duration, greater intraoperative blood loss, and higher osteotomy grade (*P < *0.05). Improvements in FVC% and FEV1% were significantly smaller in the HGT group (*P < *0.001). While both groups showed postoperative gains in SRS-22r scores, the differences between them were not statistically significant. No neurological complications occurred in either group; one case of iliac pin breakage in the HPT group was managed successfully without impacting the surgical outcome.

**Conclusion:**

Both HGT and HPT were feasible and safe in the preoperative management of patients with severe rigid spinal deformity. In this limited cohort, HPT was associated with greater angular correction, improved pulmonary function, and reduced intraoperative complexity compared to HGT. While these findings are encouraging, larger prospective studies are warranted to validate the long-term efficacy and safety of HPT and to better inform clinical decision-making in high-risk spinal deformity cases.

## Introduction

Severe rigid spinal deformity is typically defined as a spinal deformity characterized by a coronal or sagittal Cobb angle exceeding 90°, with spinal flexibility of less than 30% in the supine coronal bending test [1–4].These deformities are often associated with thoracic cage abnormalities and compromised pulmonary function, rendering corrective surgery technically demanding and high risk, with a substantial likelihood of perioperative complications [5]. Currently, surgical correction of severe rigid spinal deformity remains one of the most formidable challenges in spinal surgery [6–8]. To reduce surgical risks, preoperative traction techniques are often used to improve spinal flexibility [9, 10], decrease curve magnitude, and evaluate spinal cord tolerance, ensuring safer surgical planning [1, 11].

Currently, the primary traction techniques employed in the management of severe spinal deformities include halo-gravity traction (HGT), halo-pelvic traction (HPT), and halo-femoral traction (HFT) [12]. HPT, the most historically established approach, was first introduced by Perry and Nickel in 1959 for stabilizing unstable cervical segments [13]. In the 1970 s, O’Brien et al. refined the technique and successfully applied it to scoliosis correction, paving the way for its widespread adoption in spinal surgery [14]. HGT employs a halo ring to deliver axial gravitational traction, utilizing a progressive loading mechanism that enhances spinal flexibility while significantly reducing the risk of neurological complications [10, 15–17]. HFT, in contrast, applies traction at the supracondylar region of the femur in combination with head traction via a halo device, providing corrective forces. However, its clinical utility in severe rigid deformities is limited by its relatively low traction strength and the requirement for mandatory bed rest, as patients must remain in a strictly supine position throughout the treatment period, potentially leading to additional complications [18]. Given these considerations, HGT and HPT have emerged as the preferred traction strategies for optimizing surgical outcomes in complex spinal deformities.

Despite the widespread use of HGT and HPT, a comprehensive comparison of their efficacy in preoperative clinical improvement, surgical optimization, and psychological impact remains lacking. This study conducted a 2-year retrospective analysis of patients with severe rigid spinal deformity, comparing the advantages and disadvantages of HPT in treating complex spinal deformities, providing evidence to guide the selection of optimal traction strategies in clinical practice.

## Methods

### Study design and ethics approval

The study protocol was reviewed and approved by the Ethics Committee of the Affiliated Hospital of Qingdao University (Approval No. QDFY WZLL 30209) and the Ethics Committee of Shandong Provincial Hospital, Shandong First Medical University (Approval No. SWYX2024-417), and informed consent was obtained from all participants and their legal guardians. We conducted a retrospective analysis of patients diagnosed with severe rigid spinal deformity (Cobb angle > 90°, flexibility < 30%) who were admitted to the spinal surgery departments of The Affiliated Hospital of Qingdao University and Shandong Provincial Hospital between January 2019 and December 2022. All patients underwent preoperative HGT or HPT prior to posterior corrective surgery. Based on the method of traction, patients were categorized into the HGT or HPT group. Parameters evaluated included demographic characteristics, coronal and sagittal Cobb angles, correction rates, pulmonary function metrics, intraoperative blood loss, operative time, osteotomy grade, and SRS-22r scores during follow-up. Inclusion criteria were as follows: (i) a coronal or sagittal Cobb angle exceeding 90°; and (ii) a coronal flexibility index < 30% on supine bending radiographs. In other words, all patients were required to demonstrate a coronal flexibility index < 30%, and, on this basis, eligibility was met if either the coronal or sagittal Cobb angle exceeded 90°. Exclusion criteria included incomplete clinical data, refusal of traction treatment, or a history of prior spinal surgery. Among the 20 patients, 2 fulfilled only the coronal ≥ 90° criterion, 2 fulfilled only the sagittal ≥ 90° criterion, and the remaining 16 fulfilled both criteria. All patients satisfied the requirement of a coronal flexibility index < 30% on supine bending radiographs.

### HGT protocol

**Fig. 1:**
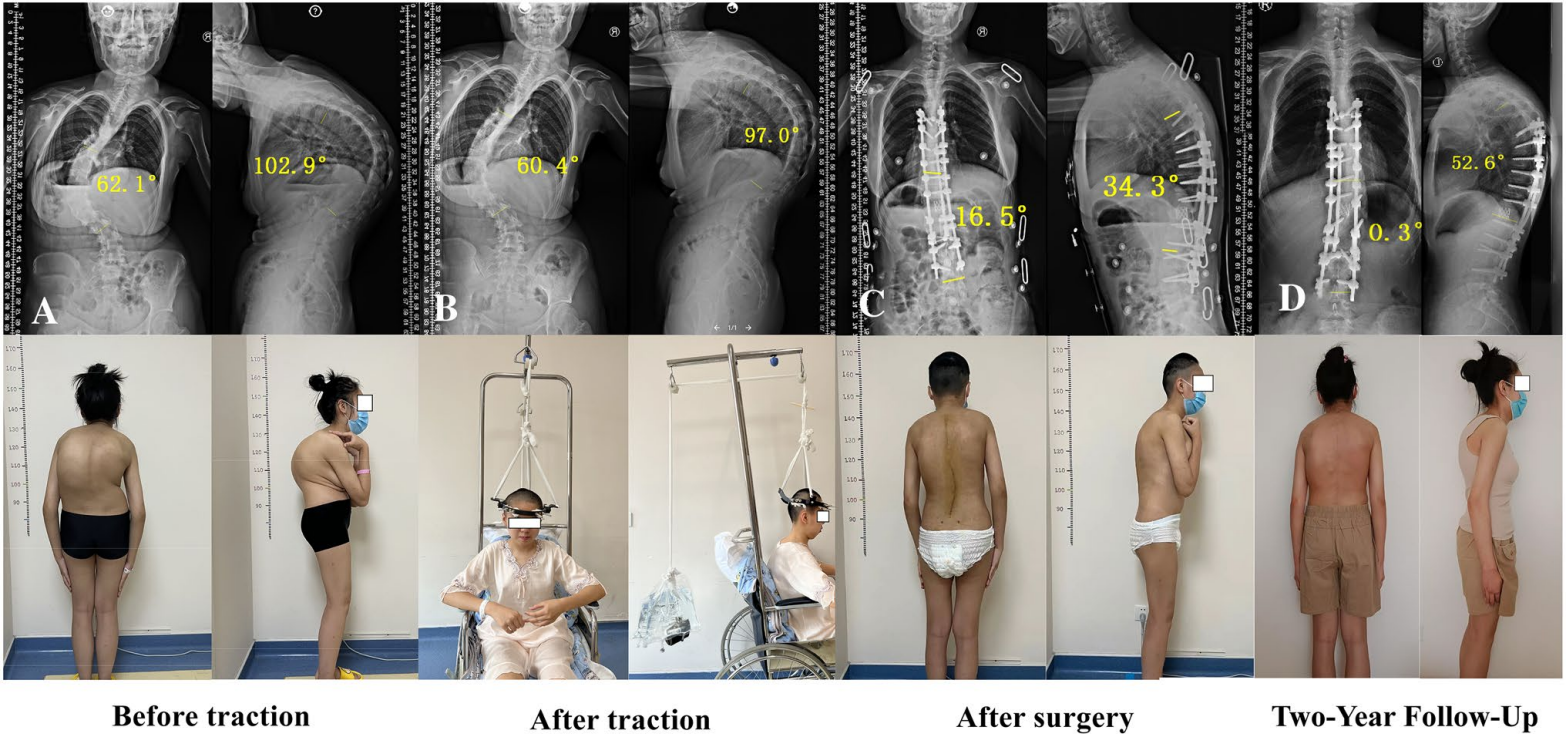
Preoperative application of HGT in a 15-year-old girl with severe rigid spinal deformity. **A** The patient's initial spinal deformity presented with a coronal scoliosis angle of 62.1° and a sagittal kyphosis angle of 102.9°. **B** After traction, the scoliosis angle reduced to 60.4° and the kyphosis angle to 97°. **C** Posterior-assisted osteotomy and fusion surgery were performed from T6 to L4, with a grade 5 osteotomy at T12 for posterior reconstruction. **D** The patient had a two-year follow-up, and no abnormalities were found. The spinal fusion appears solid with no signs of hardware loosening, breakage, or loss of correction

Under local anesthesia, the HGT apparatus was installed with the patient in a seated position. An appropriately sized halo ring was fitted to the skull, and four pins were inserted through pre-drilled holes—positioned above the lateral third of the bilateral supraorbital ridges and the posterosuperior region of the auricles—anchoring firmly into the outer cortical bone of the cranium. Traction was applied either in a wheelchair or in a standing position, with an initial force set at 20% of the patient’s body weight and gradually increased to 50% over a period of four weeks. Continuous traction was maintained throughout the treatment course, with brief interruptions permitted only for meals and personal hygiene. Pin sites were inspected daily by the surgical team for signs of infection or neurological compromise. In this study, halo-gravity traction (HGT) was applied using a pulley-based construct, with patients undergoing traction primarily in the wheelchair or seated position. During the study period, spring-based ambulatory traction systems, which are more commonly employed in contemporary practice, were not utilized (Fig. 1).

### HPT protocol

**Fig. 2:**
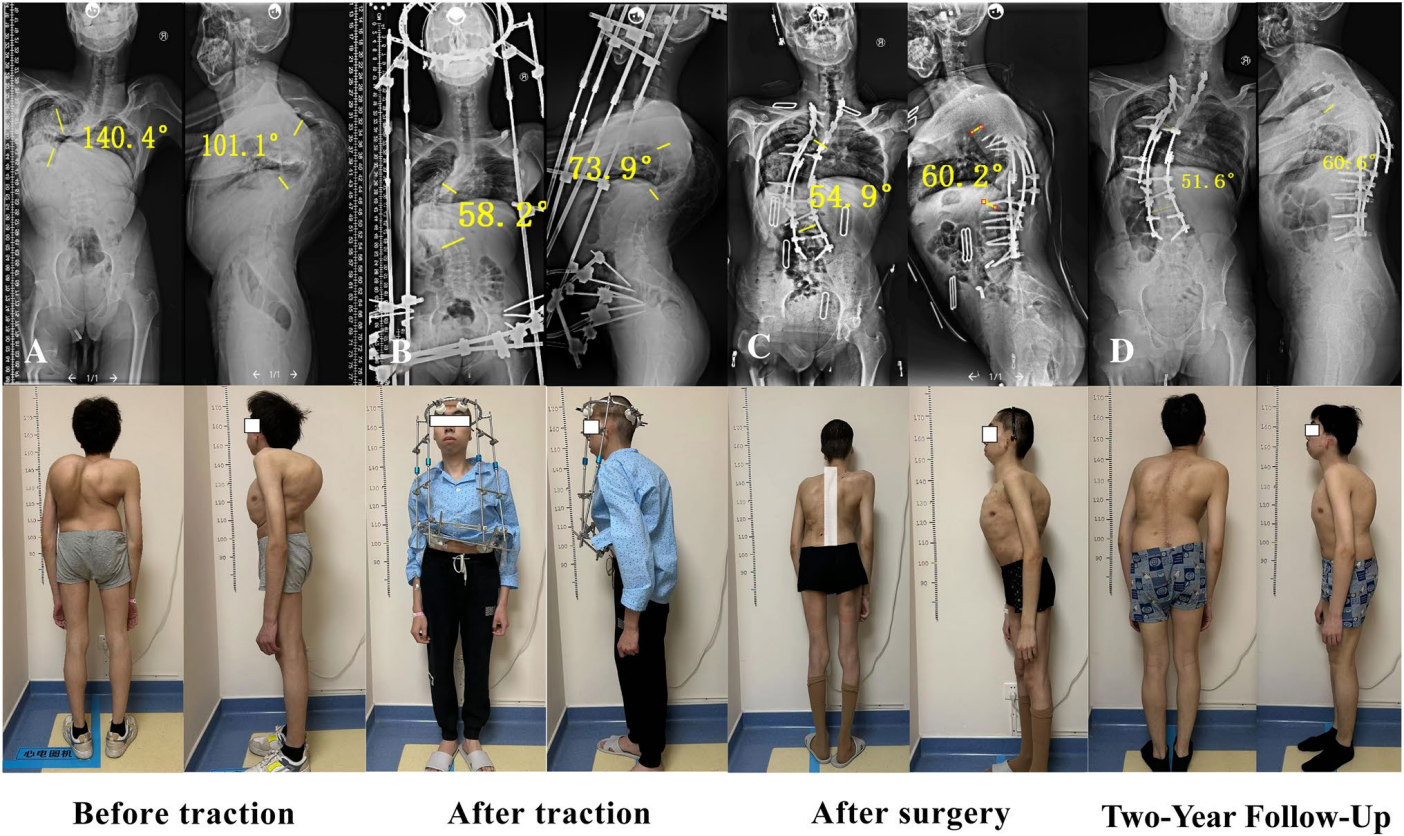
Preoperative application of HPT in a 20-year-old male with severe rigid spinal deformity. **A** The patient initially presented with a coronal scoliosis angle of 140.4°, a sagittal kyphosis angle of 101.1°, and a visibly deformed general appearance. **B** After 3 months of traction, the scoliosis angle decreased to 70.4°, and the kyphosis angle reduced to 73.9°. **C** He underwent posterior reconstruction from T1 to L5, including an asymmetric pedicle subtraction osteotomy (PSO) at T10. **D** The patient had a two-year follow-up, and no abnormalities were found. The spinal fusion appears solid with no signs of hardware loosening, breakage, or loss of correction

With the patient in the supine position, an appropriately sized halo ring was selected. Four cranial pins were inserted through designated holes in the halo ring—positioned above the bilateral superolateral supraorbital ridges, avoiding the frontal muscle, and superior to the auricles—and anchored into the outer cortical layer of the skull. According to pelvic anatomy and surgeon preference, two to three transiliac pins were inserted on each side. These pelvic pins were connected to a pelvic ring, and four traction rods were positioned between the halo and pelvic rings. Distraction was achieved by progressively rotating traction nuts on the rods. During the 1 st week, the distraction rate was set at 1–2 cm per day. From the 2nd week onward, the rate was reduced to 0.5 cm per day (Fig. 2).

### Termination time of traction

The termination time of HGT traction is not pre-set, but dynamically adjusted based on individual patient conditions. The main criteria include whether the correction of Cobb angle tends to reach a plateau in weekly imaging examinations, and whether pulmonary function and nutritional status have achieved the preoperative optimization goals. In contrast, the termination time of HPT traction is generally determined by the absence of significant progress, a marked increase in the resistance of rotating nuts, or the occurrence of intolerable neck muscle spasms in patients. Traction should be terminated immediately if brachial plexus or cranial nerve root symptoms appear. For both traction methods, corrective surgery is performed immediately after the completion of traction.

### Intraoperative traction conditions

Intraoperative traction with both HGT and HPT was continuously performed, and the same standards were maintained across all participating hospitals. Traction was sustained during soft tissue exposure and removed after pedicle screw placement. This standard was strictly adhered to in all participating hospitals to ensure consistency of treatment and comparability of outcomes.

### Radiographic and clinical evaluation

The coronal and sagittal Cobb angles before traction, after traction, postoperatively, and at the 2-year follow-up after surgery were analyzed. The spinal flexibility index was calculated using preoperative coronal bending radiographs. The flexibility index was calculated only in the coronal plane, as the bending test was performed in the supine coronal view. Sagittal flexibility was not separately quantified due to limitations in imaging protocol. Radiological evaluations were performed by senior physicians. Traction correction rate = (Cobb angle before traction − Cobb angle after traction)/Cobb angle before traction. Postoperative correction rate = (Cobb angle after traction − Cobb angle after surgery)/Cobb angle after traction. The improvement attributed to traction was evaluated based on the reduction in Cobb angles, as post-traction flexibility imaging was not performed. The change in height is represented by the difference between the height after traction and the height before traction.

### Pulmonary function tests

Pulmonary function tests (PFTs) were performed at two time points: before the initiation of traction and upon completion of the traction period. The primary parameters assessed were the percentage of predicted forced vital capacity (FVC%) and forced expiratory volume in one second (FEV1%), as well as their respective improvements, calculated as the difference between post-traction and pre-traction values. Given the potential inaccuracy of actual height measurements in patients with severe spinal deformities—due to both anatomical distortion and traction effects—arm span was used as a surrogate for height in calculating the predicted values for pulmonary function indices.

### SRS-22r scores

The SRS-22r scores assesses five domains: pain, self-image, function/activity, mental health, and satisfaction with treatment, providing a comprehensive evaluation of patients'quality of life and postoperative recovery. In this study, SRS-22r scores were recorded at four time points: preoperatively, and at 6, 12, and 24 months postoperatively. Scores for each domain were compared between the HGT and HPT groups at each time point to evaluate differences in recovery trajectories [19].

### Statistical analysis

Categorical variables were compared using the Pearson's Chi-square test. For continuous variables, after verifying normality and homogeneity of variance, two-way mixed ANOVA was applied to repeated measures indicators such as coronal/sagittal Cobb angles, pulmonary function parameters (FVC/FEV1), and SRS-22r scores to analyze the main effect of time, main effect of group, and interaction effect (TIME × GROUP), with the corresponding *P* values reported. If normality was not satisfied, nonparametric mixed models were used instead. As osteotomy grade represents an ordinal variable, between-group differences were evaluated using the Wilcoxon signed-rank test. All statistical analyses were performed using the SPSS software (SPSS Inc., Chicago, Illinois, USA), and a two-sided *P* value < 0.05 was considered statistically significant.

## Results

### Patient demographics and clinicopathological data

**Fig. 3:**
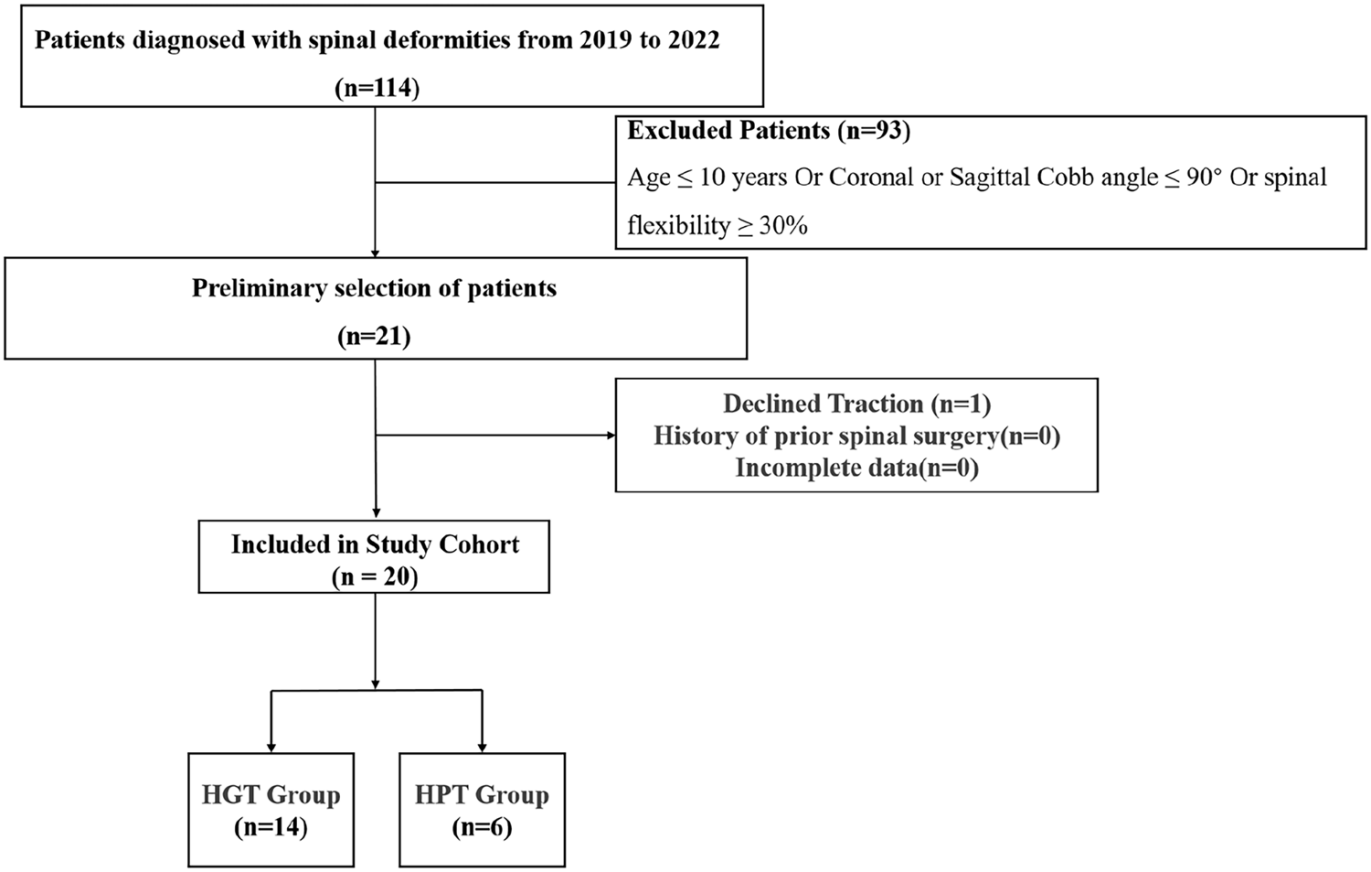
CONSORT flow diagram

From 2019 to 2022, 21 patients with scoliosis and kyphosis were screened from 114 patients diagnosed with spinal deformities in this study, meeting the following criteria: age over 10 years, a Cobb angle exceeding 90° on standing full-spine X-rays, and spinal flexibility less than 30% on the supine coronal bending test. One patient was excluded due to refusal of traction, and the remaining patients were not excluded for reasons such as incomplete case data or a history of previous spinal surgery. Eventually, 20 patients were included in the analysis (see Fig. 3). Based on the type of preoperative traction received, patients were categorized into the HGT group (*n = *14) and the HPT group (*n = *6). Baseline demographic and clinical characteristics recorded at the initial visit are summarized in Table 1. There were no statistically significant differences between groups in terms of age, sex, body mass index (BMI), or flexibility index. As shown in Table 1, the etiologies in the HGT group included idiopathic scoliosis (IS, 5 cases), congenital scoliosis (CS, 3 cases), neuromuscular scoliosis (NM, 2 cases), neurofibromatosis (NF, 3 cases), and Gaucher disease-associated scoliosis (GS, 1 case). The HPT group included two IS cases, three CS cases, and one NF case. The average duration of traction was 8 weeks in the HGT group and 5.3 weeks in the HPT group.

**Table 1:** The demographic in the HGT group and the HPT group

	HGT (*n = *14)	HPT (*n = *6)	*P* value
Age [$\overline{x} \pm s,{\text{ y}}$]	16.4 ± 3.2	17.3 ± 3.5	0.601
Sex [*n* (%)]			1
Male	8(57.14)	3(50)	
Female	6(42.86)	3(50)	
BMI [$\overline{x} \pm s,{\mathrm{kg}}/{\mathrm{m}}^{2}$]	23.9 ± 2.8	24.7 ± 2.5	0.569
Diagnosis [*n* (%)]			0.578
IS	5(35.71)	2(33.33)	
CS	3(21.43)	3(50)	
NF	3(21.43)	1(16.67)	
NM	2(14.29)	0	
GS	1(7.14)	0	
Coronal flexibility index [$\overline{x} \pm s,\%$]	14.6 ± 4.6	14.9 ± 5.9	0.915
Traction time [$\overline{x} \pm s,{\text{ week}}$]	8 ± 1.5	5.3 ± 1.0	< 0.001

*HGT* halo-gravity traction, *HPT* halo-pelvic traction, *IS* idiopathic scoliosis, *CS* congenital scoliosis, *NM* neuromuscular scoliosis, *NF* neurofibromatosis, GS Gaucher disease-associated scoliosis

### Radiographic evaluation

The coronal Cobb angle showed a significant interaction effect between the time and traction method (TIME × GROUP *P* = 0.032), indicating that the correction effect of the Cobb angle differs significantly between the HGT and HPT methods at different time points. However, in the postoperative phase, the correction effects of HGT and HPT were similar (GROUP *P* = 0.218). Similarly, the sagittal Cobb angle exhibited comparable results (TIME × GROUP *P* = 0.028). There was no significant difference in the effects between the two groups postoperatively (GROUP *P* = 0.235) (Table 2).

**Table 2:** Mixed analysis of variance for Coronal Cobb angle and Sagittal Cobb angle

Parameter	TIME × GROUP, *P*	TIME, *P*	GROUP, *P*	Mauchly’s test
Coronal Cobb angle	0.032	< 0.001	0.218	G-G ε = 0.87
Sagittal Cobb angle	0.028	< 0.001	0.235	G-G ε = 0.89

There was no significant difference in the mean pre-traction Cobb angles between the two groups. At the end of the traction period, the mean coronal Cobb angle was reduced to 79 ± 14° in the HGT group and 66 ± 14° in the HPT group, corresponding to average corrections of 32 ± 12° and 50 ± 16°, respectively (*P* = 0.006). Following definitive corrective surgery, the mean coronal Cobb angles further decreased to 54 ± 18° in the HGT group and 52 ± 14° in the HPT group. No significant difference was found in the pre-traction sagittal Cobb angles between groups. By the end of traction, the sagittal Cobb angle was reduced to 80 ± 17° in the HGT group and 69 ± 15° in the HPT group, with average correction percentages of 29 ± 14% and 39 ± 14%, respectively (*P < *0.001). Postoperatively, the sagittal Cobb angles were corrected to 61 ± 14° in the HGT group and 58 ± 16° in the HPT group. During the 2-year postoperative follow-up, no significant progression of deformity was observed in either group. Additionally, average height gain during traction was significantly greater in the HPT group (13.3 ± 1.7 cm) compared to the HGT group (10.1 ± 1.9 cm) (*P* = 0.002). Detailed results are presented in Table 3.

**Table 3:** Comparison of imaging parameters and growth height between the HGT group and HPT group

	HGT (*n = *14)	HPT (*n = *6)	*P* value
Coronal Cobb angle [$\overline{x} \pm s,{ }^\circ$]			
Pre-traction	111 ± 16 (62–140)	116 ± 15 (92–150)	0.681
Post-traction	79 ± 14 (60–98)	66 ± 14 (48–88)	0.029
Post-surgery	54 ± 18 (35–92)	52 ± 14 (36–74)	0.133
2-year follow-up	55 ± 17 (36–94)	52 ± 16 (34–76)	0.248
Traction correction	32 ± 21	50 ± 20	NA
Post-surgery correction	25 ± 22	14 ± 19	NA
Sagittal Cobb angle [$\overline{x} \pm s,{ }^\circ$]			
Pre-traction	109 ± 17 (78–145)	108 ± 16 (82–142)	0.714
Post-traction	80 ± 17 (62–115)	69 ± 15 (50–92)	< 0.001
Post-surgery	61 ± 14 (38–88)	58 ± 16 (35–82)	0.645
2-year follow-up	62 ± 13 (40–84)	59 ± 10 (42–75)	0.532
Traction correction	29 ± 24	39 ± 21	NA
Post-surgery correction	19 ± 22	11 ± 21	NA
Growth height [$\overline{x} \pm s,{\mathrm{CM}}$]	10.1 ± 1.9	13.3 ± 1.7	0.002

*HGT* halo-gravity traction, *HPT* halo-pelvic traction

### Operative parameters

As shown in Table 4, the HGT group was more Likely to undergo higher-level osteotomy. In terms of osteotomy location, procedures were concentrated in the thoracolumbar region. In the HGT group, grade 5 osteotomies (VCRs, 4 cases) were predominantly performed at thoracic levels T7–T10, while grade 3 osteotomies (PSOs, 4 cases) were located mainly at lumbar levels L1–L3. Additionally, three grade 4 and three grade 2 osteotomies were distributed around the thoracolumbar junction (T11–L2). In the HPT group, most procedures were PSOs at L1–L3 (4 cases), with one grade 4 osteotomy at T12–L1 and one grade 2 osteotomy at T11–T12. Overall, curve apices were located between T8 and L2. There was a significant statistical difference in intraoperative blood loss between the two groups: the HGT group had an intraoperative blood loss of 632.14 ± 106.71 cc (15.2 ± 3.1% EBV), while the HPT group had 483.33 ± 103.28 cc (11.5 ± 2.7% EBV). Additionally, the intraoperative surgery time was significantly different between the HGT and HPT groups (5.07 ± 1.07 vs. 3.83 ± 0.75, *P* = 0.011).

**Table 4:** Comparison of surgical indexes between HGT group and the HPT group

	HGT (*n = *14)	HPT (*n = *6)	*P* value
Surgical bleeding [$\overline{x} \pm s,{\text{ cc}}$]	632.1 ± 106.7	483.3 ± 103.3	0.015
Surgical bleeding [$\overline{x} \pm s,$%EBV]	14.6 ± 4.6	11.2 ± 3.9	
Operation time [$\overline{x} \pm s,{\text{ h}}$]	5.1 ± 1.1	3.8 ± 0.8	0.011
Osteotomy grade [*n* (%)]			0.044
6	0	0	
5	4	0	
4	3	1	
3	4	4	
2	3	1	
1	0	0	

Osteotomy grades in this table follow the Lenke classification system

*HGT* halo-gravity traction, *HPT* halo-pelvic traction

### Pulmonary function

The improvement in FVC and FEV1 was significantly better in the HPT group compared to the HGT group (FVC: GROUP p = 0.025; FEV1: GROUP *P* = 0.028). Furthermore, the main effect of time was significant in the improvement of both FVC and FEV1 (TIME *P < *0.001) (Table 5). There were no significant differences in the baseline pulmonary function parameters between the two groups, in terms of FVC% (HGT group: 41.2 ± 2.9, HPT group: 41.9 ± 2.8, *P* > 0.05), and FEV1% (HGT group: 44.3 ± 5.9, HPT group: 53.9 ± 5.9, P > 0.05). At the end of traction, the FVC% in the HGT group was corrected to an average of 47.3 ± 1.1, while in the HPT group it was 60.9 ± 3.7 (*P < *0.001). The mean improvement in FVC% was 6.1 in the HGT group and 19.0 in the HPT group. Similarly, the mean improvement in FEV1% was 2.0 in the HGT group and 6.0 in the HPT group (*P < *0.001). The improvement of FEV1% was weaker in the HGT group compared to the HPT group, with a significant difference between the two groups (*P < *0.001). Pulmonary function data are shown in Table 6.

**Table 5:** Mixed analysis of variance for FVC and FEV1

Parameter	TIME × GROUP, *P*	TIME, *P*	GROUP, *P*	Mauchly’s test
FVC	0.045	< 0.001	0.025	Sphericity Assumed
FEV1	0.032	< 0.001	0.028	Sphericity Assumed

**Table 6:** Pulmonary function testing results for patients before and at the end of traction

	HGT (*n = *14)	HPT (*n = *6)	*P* value
FVC [$\overline{x} \pm s,{\text{ \% }}$]			
Pre-traction	41.2 ± 2.9	41.9 ± 2.8	0.647
End of the traction phase	47.3 ± 1.1	60.9 ± 3.7	< 0.001
FEV1 [$\overline{x} \pm s,{\text{ \% }}$]			
Pre-traction	44.3 ± 5.9	53.9 ± 5.9	0.818
End of the traction phase	46.3 ± 1.9	59.9 ± 1.7	< 0.001

*HGT* halo-gravity traction, *HPT* halo-pelvic traction

### SRS-22r scores

In both the HGT and HPT groups, SRS-22r scores across all five domains—pain, self-image, function/activity, mental health, and satisfaction with treatment—showed improvements at 6, 12, and 24 months postoperatively compared to preoperative baseline. However, no statistically significant differences were observed between the two groups at any of the assessed time points (Table 7).

**Table 7:** Mixed analysis of variance for the SRS-22r scores

Parameter	TIME × GROUP, *P*	TIME, *P*	GROUP, *P*	Mauchly’s test
SRS-22r scores	0.078	< 0.001	0.651	Sphericity Assumed

### Complications

The HGT group had no pin tract infections, screw fractures, or loosening of fixation devices during the treatment period. In the HPT group, one patient experienced a fatigue fracture of the iliac screw after 10 weeks of traction. Neither group developed neurological deficits or deep surgical site infections during the postoperative follow-up. The screw fracture in the HPT group was managed with an emergency replacement of the pelvic fixation module, allowing continued traction without affecting the final surgical outcome.

## Discussion

Surgical treatment of severe rigid spinal deformity involves significant challenges, particularly in deformity correction and spinal cord protection. Preoperative traction improves spinal flexibility and pulmonary function, reducing surgical risks [20]. Although the two main traction techniques, HGT and HPT, are commonly used, there is a lack of systematic comparison regarding their preoperative clinical effectiveness, optimization of surgical conditions, and psychological impact. By analyzing clinical data from 20 patients with severe rigid spinal deformity and integrating both observational findings and clinical experience, this study aimed to assess the relative advantages and limitations of HGT and HPT, providing a reference for future prospective studies with larger sample sizes to validate long-term efficacy and safety.

This study used mixed analysis of variance and found a significant interaction effect between time and traction method on the coronal and sagittal Cobb angles, indicating that the traction method has a significant impact on the correction of the Cobb angle at different time points. Specifically, the HPT group showed a significant correction effect on the Cobb angle after traction, demonstrating that HPT is more effective during the traction process. However, in the postoperative phase, the correction effects of HGT and HPT became similar, which may be due to the fact that the degree of osteotomy in the HGT group was significantly higher than that in the HPT group, compensating for the shortcomings during the traction phase and achieving a similar final correction effect to the HPT group. However, patients in the HPT group, due to larger residual deformity angles or severe spinal stiffness, still required three-column osteotomy (3CO) during surgery to complete the final correction. White and Panjabi have suggested that curves exceeding 60° already warrant traction evaluation to assess spinal flexibility [21]. In contrast, our cohort only included patients with curves greater than 90° and flexibility below 30%, representing a more severe subset of deformities. This difference may partly explain the higher traction correction demands observed in our study population.

This study found that both the HGT and HPT groups exhibited significant improvements in coronal and sagittal Cobb angles following traction, underscoring the value of preoperative traction in the correction of severe spinal deformities. However, further analysis revealed that the HPT group achieved significantly greater angular correction at the end of the traction period and required a shorter duration of traction, suggesting superior corrective efficacy. Additionally, both traction methods contributed to improved spinal flexibility and a reduced need for high-grade osteotomies during surgery. Notably, the HPT group underwent less extensive osteotomies compared to the HGT group, likely due to the higher mechanical force generated by the pelvic fixation system, which more effectively improved spinal pliability.

The statistical results of this study show that the main effect of time is significant in the improvement of both FVC and FEV1 (*P < *0.05), suggesting that both traction methods may have a positive effect on improving pulmonary function. Further analysis revealed that the HPT group showed greater improvement in FVC and FEV1 at the end of traction compared to the HGT group (inter-group comparison *P < *0.05). It is important to note that the above conclusions are based only on the measurements taken at the end of the traction phase. Since this study did not collect post-operative and 2-year follow-up PFT data, it is currently unclear whether HPT has a long-term advantage in improving pulmonary function. Considering the correlation between Cobb angles and PFTs, the difference in Cobb angles between the two groups at the end of the traction phase (with more significant correction in the HPT group) may diminish as the postoperative correction effects converge. However, this hypothesis still requires validation through postoperative data in future studies. A retrospective study by Liang et al. [22] of 37 patients with severe rigid scoliosis who underwent preoperative traction with HPT showed that HPT significantly improved FVC and FEV1, which is consistent with the results of this study, where “the HPT group showed more significant pulmonary function improvement at the end of traction.” Based on existing data and supporting similar studies, the effect of HPT on improving pulmonary function during the preoperative traction phase warrants attention. However, its application value in the treatment of severe spinal deformities still requires further clarification through studies with larger sample sizes and longer follow-up periods.

Additionally, the intraoperative blood loss in the HGT group was significantly higher than that in the HPT group, suggesting that HPT may have a potential advantage in reducing the risk of intraoperative bleeding. Compared to the HPT group, the average surgical time for the HGT-assisted posterior osteotomy was longer, mainly due to the higher osteotomy level and greater blood loss in the HGT group. This not only increased the complexity of the osteotomy procedures but also prolonged the overall surgical duration. Overall, this study preliminarily shows that HPT has certain advantages in correction efficiency during the traction phase and control of intraoperative blood loss, providing initial reference for the selection of traction methods. However, due to the small sample size (HPT group *n = *6), its clinical value needs to be validated in studies with larger sample sizes.

The findings of this study are broadly consistent with those reported in the meta-analysis by Sun et al. [1], further supporting the efficacy of HPT traction in the treatment of severe rigid spinal deformity. However, the magnitude of Cobb angle correction observed in the HPT group in this study was slightly lower than that reported in the meta-analysis (coronal: 50° vs. 57°; sagittal: 39° vs. 45°). This discrepancy may be attributed to factors such as variability in spinal flexibility, surgeon preference, and the relatively small sample size of this two-center cohort. Additionally, as a two-center retrospective study with small sample size, the present work may not fully reflect the heterogeneity of patient populations included in large-scale meta-analyses. Importantly, the meta-analysis also demonstrated that HPT traction significantly improves pulmonary function parameters, including FVC and FEV1—findings that are corroborated by our results and further underscore the positive role of HPT in preoperative optimization.

This study also evaluated the psychological state and quality of life of patients at different stages. The analysis results indicated a significant main effect of time, suggesting that the patients'quality of life improved significantly over the course of traction treatment. However, there was no significant difference between the traction methods, indicating that regardless of the traction method used, the effect on improving quality of life was similar.

However, despite the favorable outcomes associated with HPT, its clinical application is not without limitations, which warrant careful consideration in practice. During traction, patients may experience restricted mouth opening, difficulty in eating, and limited lower abdominal mobility. Additionally, the mechanical forces exerted by the device may pose a higher risk of traction-related complications involving cranial nerves and the brachial plexus. Moreover, the HPT apparatus relies on stable pelvic fixation, rendering it unsuitable for patients with incomplete pelvic development—such as younger adolescents—or those with severe osteoporosis, thereby limiting its applicability in certain populations. As such, when selecting a treatment strategy, the corrective advantages of HPT should be carefully balanced against its potential risks.

This study has several Limitations. First, the small sample size Limits the generalizability and statistical power of the findings. Second, the 24-month follow-up may be insufficient to evaluate long-term outcomes, such as correction durability and adjacent segment degeneration. Extending follow-up to 5 years would improve the clinical relevance of future studies. Third, although we reported the general distribution of osteotomy locations and curve apices, more detailed vertebral-level analysis was not performed. This lack of granularity may have influenced the interpretation of differences in osteotomy grades between groups. Fourth, our inclusion criteria were restricted to curves > 90° with flexibility < 30%, whereas White and Panjabi have recommended that traction evaluation should already be considered for curves exceeding 60°. Therefore, our findings may not be generalizable to patients with curves between 60° and 90°, and further studies including this population are warranted. Finally, in this study, HGT was applied using a pulley-based wheelchair construct rather than the spring-based ambulatory systems that are more widely adopted in contemporary practice, which may limit the generalizability of our results to centers employing spring-based systems.

In summary, compared to HGT, HPT achieved more robust deformity correction within a shorter traction duration. By enhancing spinal flexibility, improving cardiopulmonary function, and reducing the need for high-grade osteotomies, HPT effectively minimized intraoperative blood loss and significantly shortened operative time. These advantages make HPT particularly suitable for patients with severe rigid spinal deformity complicated by cardiopulmonary dysfunction. As a valuable preoperative strategy to improve spinal pliability, HPT may contribute meaningfully to reducing overall surgical risk in this high-risk population.

## Data Availability

The datasets generated during and/or analysed during the current study are available from the corresponding author on reasonable request.
